# 12-Lipoxygenase Inhibition on Microalbuminuria in Type-1 and Type-2 Diabetes Is Associated with Changes of Glomerular Angiotensin II Type 1 Receptor Related to Insulin Resistance

**DOI:** 10.3390/ijms17050684

**Published:** 2016-05-06

**Authors:** Hong-Zhao Xu, Yan-Li Cheng, Wan-Ning Wang, Hao Wu, Yuan-Yuan Zhang, Chong-Sen Zang, Zhong-Gao Xu

**Affiliations:** Department of Nephrology, the First Hospital of Jilin University, Changchun 130021, China; xhz198906@163.com (H.-Z.X.); leoai918@163.com (Y.-L.C.); wangwn11@mails.jlu.edu.cn (W.-N.W.); docwuhao@sina.com (H.W.); zhangyuanyuan_1214@163.com (Y.-Y.Z.); renalzangchongsen@163.com (C.-S.Z.)

**Keywords:** 12-lipoxygenase, insulin resistance, angiotensin II type 1 receptor, diabetic nephropathy

## Abstract

(1) Background: 12-lipoxygenase (12-LO) is involved in the development of diabetic nephropathy (DN). In the present study, we investigated whether 12-LO inhibition may ameliorate type-2 DN (T2DN) by interfering with insulin resistance (IR); (2) Methods: Rat glomerular mesangial cells, glomeruli and skeletal muscles were isolated and used in this study. Kidney histological changes were confirmed by periodic-acid Schiff staining; mRNA expression was detected by competitive reverse transcription polymerase chain reaction; and the protein level was determined by Western blot and the enzyme-linked immunosorbent assay, respectively; (3) Results: The inhibition of 12-LO attenuated microalbuminuria (MAU) increases in type-2 diabetic rats, but not in type-1 diabetic rats. Infusion of 12(S)-hydroxyeicosatetraenoic acid (12(S)-HETE) significantly increased the expression of angiotensin II (Ang II) and Ang II type 1 receptor (AT1R), but decreased the expression of AT1R-associated protein (ATRAP) in rat glomeruli, compared to the control. An *in vitro* study revealed that both 12(S)-HETE and insulin upregulated AT1R expression in rat mesangial cells. In the presence of p38 mitogen-activated protein kinase (MAPK) inhibitor, SB202190, the 12(S)-HETE-induced ATRAP reduction was significantly abolished. Interestingly, 12-LO inhibition did not influence AT1R expression in type-1 diabetic rats, but significantly abolished the increased AT1R and Ang II expression in glomeruli of type-2 diabetic rats. Furthermore, the inhibition of 12-LO significantly corrected impaired insulin sensitivity and fast serum insulin level, as well as the p-AMP-activated protein kinase (AMPK) reduction in skeletal muscle of type-2 diabetic rats; (4) Conclusion: The inhibition of 12-LO potentially ameliorated MAU by preventing IR through the downregulation of glomerular AT1R expression in T2DN.

## 1. Introduction

Type-1 diabetes results from an autoimmune attack of insulin-producing pancreatic β-cells, while type-2 diabetes is mainly due to insulin resistance (IR) [1]. Recent studies have focused on type-2 diabetic nephropathy (T2DN), which is characterized by the development of IR and microalbuminuria (MAU) in the early period [2,3]. IR, an independent risk factor of MAU, has emerged as the direct cause of hyperglycemia in type-2 diabetes [4]. IR may exist with MAU before the onset of diabetes and lead to pathophysiological changes in the kidney [4,5]. Unlike insulin-dependent therapies for type-1 diabetes, the treatment of type-2 diabetes is focused on IR interference to correct hyperglycemia and hyperinsulinemia.

A previous study has reported that the dysregulation of the renin-angiotensin system (RAS) in metabolic syndrome favors type-2 diabetes [6]. Angiotensin II (Ang II) is an important mediator of DN in addition to the effect of high glucose *per se*. Several lines of clinical and experimental data have also supported the contribution of Ang II to the development of IR, islet β-cell dysfunction and glucose intolerance [7,8,9]. Ang II type 1 receptor (AT1R), as one of the key mediators of Ang II physiological action, is also related to the pathogenesis of IR [10]. Thus, Ang-converting enzyme inhibitor (ACEI) and Ang II type 1 receptor blocker (ARB) are currently the standard treatment for T2DN, the function of which is to diminish proteinuria and promote insulin sensitivity [11,12,13].

Lipoxygenases (LO) are a group of non-heme iron enzymes that insert molecular oxygen into 1, 4-*cis*, *cis*-pentadiene containing polyunsaturated fatty acids. These are classified as 5-, 8-, 12- and 15-LO, according to the carbon atom of arachidonic acid, in which oxygen is inserted. The activation of 12-LO leads to the formation of oxidized lipids, such as 12(S)-hydroxyeicosatetraenoic acid (12(S)-HETE) [14,15,16]. The 12-LO along with 12(S)-HETE have been reported to play critical roles in renal disorders [10,17,18,19,20,21].

Recently, several studies have demonstrated that the upregulation of AT1R in kidneys is associated with the 12-LO pathway and 12(S)-HETE [20,21]. Furthermore, 12-LO has also been shown to play important roles in the development of DN [22,23]. Nunemaker *et al.* discovered that 12-LO contributes to high fat diet-induced IR in mice [24]. A previous study also revealed that 12-LO participates in β-cell de-differentiation in human islets [25]. The 12-LO also plays important roles in metabolic stress-induced dysfunction in islet β-cells, and 12-LO activity in the islet itself is sufficient to induce whole-body glucose intolerance under conditions of high fat diet consumption [26,27]. High fat-fed 12-LO knockout mice demonstrated reduced IR and decreased macrophage infiltration into adipose tissues [28]. The expression of AT1R was upregulated in type-2 diabetic glomeruli [20], but downregulated in type-1 diabetic glomeruli [29]. Interestingly, the inhibition of 12-LO could evidently ameliorate MAU in type-2 diabetic animal models [18], but this inhibition could not reduce MAU under type-1 diabetic conditions [22]; however, the underlying mechanism remains unelucidated. Given that IR is the major distinction between type-1 and type-2 diabetes, we hypothesized that 12-LO inhibition may ameliorate T2DN proteinuria and decrease AT1R expression by interfering with IR.

## 2. Results

### 2.1. Roles of 12-LO on Fasting Blood Glucose and MAU in Type-1 Diabetic Models

**Figure 1:**
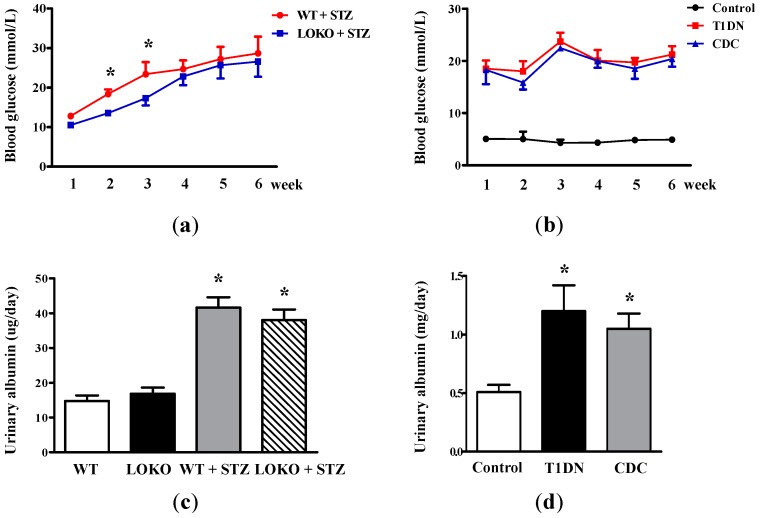
Effect of 12-LO on fasting blood glucose and MAU in type-1 diabetic models. (**a**) Fasting blood glucose levels in WT, 12-LO knockout (12-LOKO), WT + STZ and LOKO + STZ mice; *n* = 9 in each group; * *p* < 0.01 *vs.* LOKO + STZ; (**b**) fasting blood glucose levels in control, type-1 diabetic rats and CDC-treated rats; *n* = 10 in each group; (**c**) the level of MAU in WT, LOKO, WT + STZ and LOKO + STZ mice; *n* = 9 in each group; * *p* < 0.01 *vs.* WT or LOKO; (**d**) the level of MAU in control, type-1 diabetic rats and CDC-treated rats; *n* = 10 in each group. Data are presented as the mean ± standard deviation (SD); * *p* < 0.01 *vs.* the control.

In order to investigate the impact of 12-LO on fasting blood glucose level and MAU, streptozotocin (STZ) was first employed to Sprague-Dawley rats and 12-LO knockout C57BL/6 (12-LOKO) mice, as well as wild-type C57BL/6 (WT) mice to induce type-1 diabetes. Rats or mice that failed to develop hyperglycemia were excluded from this study. As shown in Figure 1a, fasting blood glucose levels in WT + STZ significantly increased compared to LOKO + STZ (*p* < 0.01) at the second and third week after STZ administration. Although fasting blood glucose of LOKO + STZ was slightly lower compared to WT + STZ during the fourth–sixth week, no significant difference was detected between these two groups; suggesting that the deletion of 12-LO slightly protected STZ-induced hyperglycemia in the early stage of diabetes. 12-LO inhibitor, cinnamyl-3,4-dihydroxy-α-cynanocinnamate (CDC), treatment slightly decreases blood glucose in STZ-induced type-1 diabetic rats compared to non-treated type-1 diabetic rats at the second and third week after STZ administration, but this did not reach statistical significance (Figure 1b). These results suggest that 12-LO inhibition may slightly decrease STZ-induced hyperglycemia for a short time by β-cell protection; nonetheless, this protective effect was gradually overcome by the destructive effect of STZ.

Figure 1c revealed that MAU significantly increased in WT + STZ and LOKO + STZ mice compared to WT mice and LOKO mice, respectively (*p* < 0.01), and the levels of MAU in LOKO + STZ and WT + STZ mice were similar; suggesting that 12-LO knockout could not prevent STZ-induced MAU elevation. Similarly, the level of MAU significantly increased in type-1 diabetic rats compared to the control (*p* < 0.01, Figure 1d); and CDC treatment did not ameliorate type-1 diabetes-induced MAU.

### 2.2. Kidney Hypertrophy, Glomerular 12(S)-HETE Levels, Fasting Blood Glucose and MAU in Type-2 Diabetic Rats

**Figure 2:**
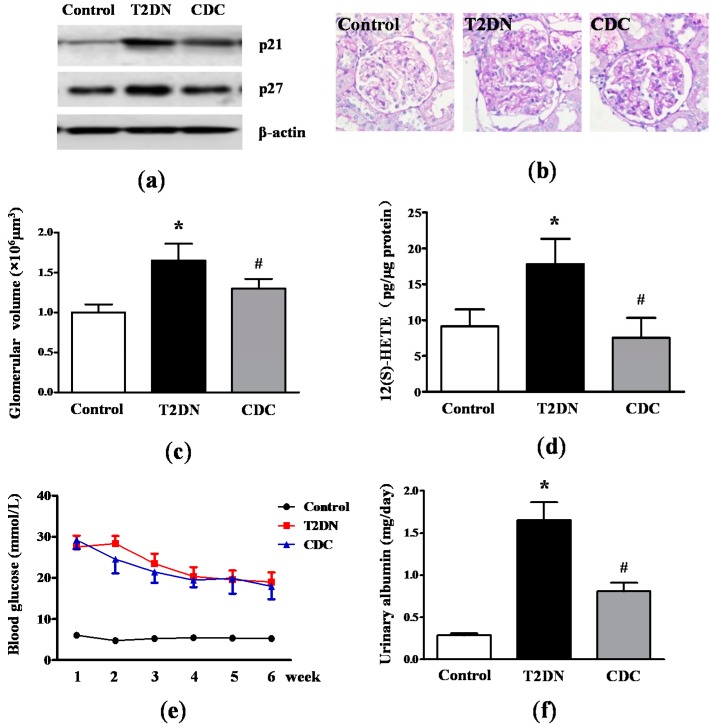
Effect of 12-LO on kidney hypertrophy, fasting blood glucose and MAU in type-2 diabetic rats. (**a**) Expression levels of p21 and p27 in glomeruli of control, type-2 diabetic rats and CDC-treated rats were determined by Western blot; (**b**) PAS staining of kidney cortical tissue in control, type-2 diabetic rats and CDC-treated rats; (**c**) glomerular volume of control, type-2 diabetic rats and CDC-treated rats; (**d**) glomerular 12(S)-HETE levels of control, type-2 diabetic rats and CDC-treated rats were measured by ELISA; (**e**) the level of fasting blood glucose in control, type-2 diabetic rats and CDC-treated rats; (**f**) the level of MAU in control, type-2 diabetic rats and CDC-treated rats; *n* = 20 in each group. Data are presented as the mean ± SD of three independent experiments; * *p* < 0.01 *vs.* the control; ^#^ *p* < 0.05 *vs.* T2DN.

Glomerular hypertrophy is one of the most striking characteristics of DN. p21 and p27, known as the Cip/Kip family, are hallmarks of kidney hypertrophy [30]. By Western blotting analysis, results revealed that p21 and p27 levels in glomeruli in type-2 diabetic rats significantly increased compared to the control (Figure 2a). Interestingly, CDC treatment dramatically reduced p21 and p27 expression. In addition, periodic acid-Schiff (PAS) staining demonstrated a remarkable enlargement of the glomeruli with mesangial expansion in type-2 diabetic rats compared to control; while these changes were significantly attenuated in rats that received CDC (Figure 2b). In order to quantify glomerular size in the different groups, glomerular volume was calculated. As shown in Figure 2c, the glomerular volume of diabetic rats was significantly higher than control (*p* < 0.01), and this change was also ameliorated by CDC treatment (*p* < 0.05). Glomerular 12(S)-HETE levels were determined by the enzyme-linked immunosorbent assay (ELISA). We could observe from Figure 2d that glomerular 12(S)-HETE levels increased significantly in type-2 diabetic rats compared to the control (*p* < 0.01). However, CDC treatment significantly attenuated the abnormality (*p* < 0.05). Next, the fasting blood glucose level was examined in type-2 diabetic rats for six weeks. It was found that CDC treatment could partially attenuate fasting blood glucose in diabetic rats compared to non-CDC treated type-2 diabetic rats at the second and third week, but this decrease was not statistically significant (Figure 2e). Furthermore, a significant elevation in MAU was also observed in type-2 diabetic rats compared to the control (*p* < 0.01), and CDC treatment notably ameliorated the increased MAU (*p* < 0.05; Figure 2f).

### 2.3. Roles of 12-LO on IR-Related Parameters in Type-2 Diabetic Rats

**Figure 3:**
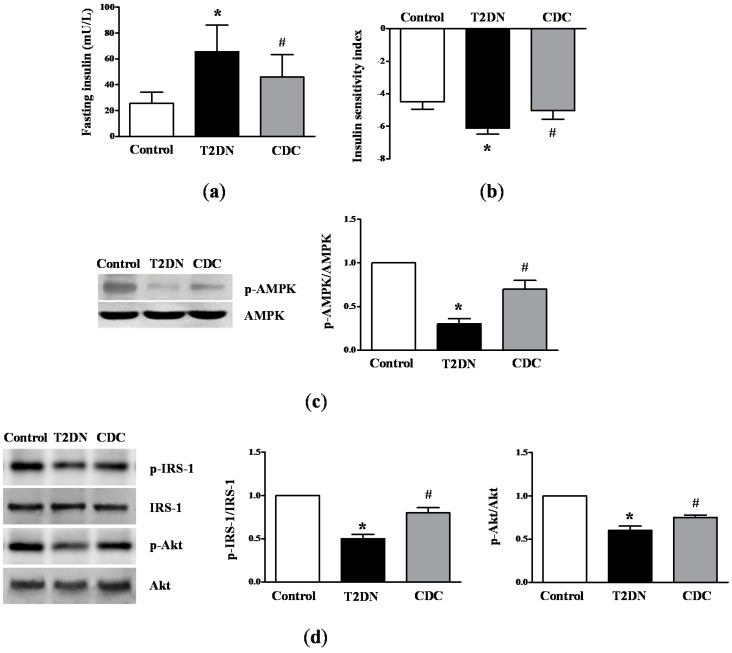
Effect of 12-LO on IR-related parameters in type-2 diabetic rats. (**a**) Fasting insulin levels were determined by radioimmunoassay (RIA); (**b**) ISI was measured as 1/(fasting insulin × fasting glucose); (**c**) AMPK expression in skeletal muscle was determined by Western blot; *n* = 20 in each group; (**d**) p-IRS-1, IRS-1, p-Akt and Akt levels in skeletal muscle were determined by Western blot; *n* = 20 in each group. Data are presented as the mean ± SD of three independent experiments; * *p* < 0.01 *vs.* the control; ^#^ *p* < 0.05 *vs.* T2DN.

In order to evaluate the effect of 12-LO on IR-related parameters, fasting insulin level, insulin sensitivity index (ISI) and AMP-activated protein kinase (AMPK) activation were measured. As shown in Figure 3a, a significant increase in fasting insulin level was observed in diabetic rats compared to control rats (*p* < 0.01); and CDC treatment prevented the diabetes-induced increase in fasting insulin level (*p* < 0.05). Furthermore, it was found that ISI was significantly lower in diabetic rats compared to the control (*p* < 0.01), and CDC treatment attenuated this abnormality (*p* < 0.05; Figure 3b). Furthermore, a significant decrease in p-AMPK level in skeletal muscles was observed in diabetic rats, compared to the control (*p* < 0.01); and CDC treatment partially, but significantly prevented the decrease in p-AMPK levels (*p* < 0.05; Figure 3c). We also detected the role of 12-LO in the insulin signaling insulin receptor substrate (IRS)-protein kinase B (Akt) pathway. As shown in Figure 3d, we found that p-IRS-1 and p-Akt levels decreased significantly in skeletal muscle of type-2 diabetic rats compared to the control (*p* < 0.01), and CDC treatment ameliorated the abnormalities (*p* < 0.05). These results indicate that CDC administration improved IR.

### 2.4. Effect of 12(S)-HETE on Rat Blood Glucose and Blood Pressure

Rats were given a subcutaneous infusion of 12(S)-HETE with osmotic mini-pumps for seven days. Treatment with 12(S)-HETE at the dose administered in this study resulted in no significant elevation in blood glucose (5.34 ± 0.37 mmol/L) compared to the control (4.93 ± 0.61 mmol/L). Similarly, direct 12(S)-HETE stimulation did not alter the systolic blood pressure (120.4 ± 4.7 mmHg) significantly compared to the control (117.3 ± 5.3 mmHg).

### 2.5. Effect of 12-LO on Ang II Level in Rat Glomeruli

**Figure 4:**
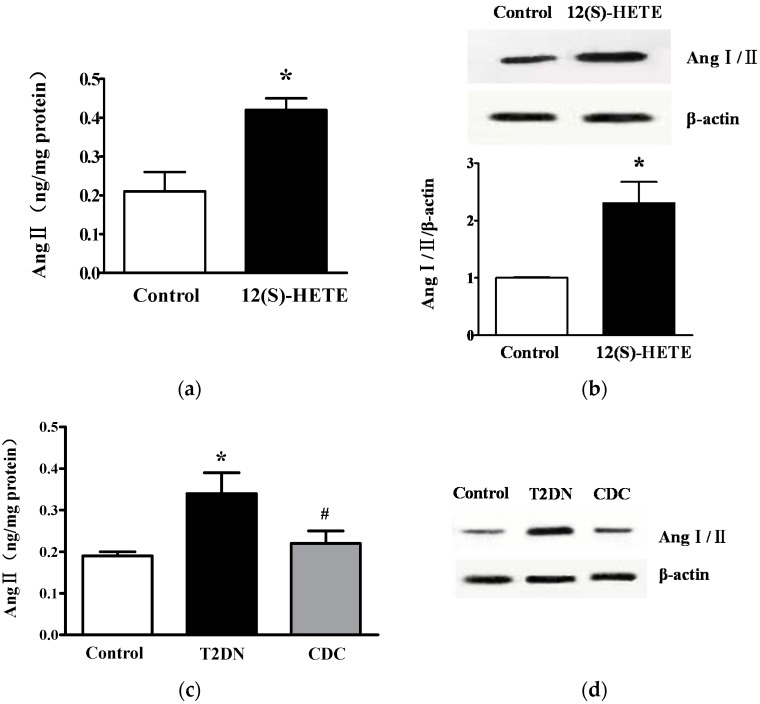
Effect of 12-LO on Ang II levels in rat glomeruli. (**a**) Ang II content in glomeruli of control and 12(S)-HETE infusion rats was determined by ELISA; *n* = 10 in each group; (**b**) Ang I/II expression in glomeruli of control and 12(S)-HETE infusion rats was determined by Western blot; *n* = 10 in each group; (**c**) Ang II content in glomeruli of control, type-2 diabetic rats and CDC-treated rats was determined by ELISA; *n* = 20 in each group; (**d**) Ang I/II expression in glomeruli of control, type-2 diabetic rats and CDC-treated rats was determined by Western blot; *n* = 10 in each group. Data are presented as the mean ± SD of three independent experiments; * *p* < 0.01 *vs.* corresponding controls; ^#^ *p* < 0.05 *vs.* T2DN.

In order to further evaluate the roles of 12-LO, the direct effect of 12(S)-HETE on Ang II level in rat glomeruli was examined. Rats were given a subcutaneous infusion of 12(S)-HETE with osmotic mini-pumps for seven days. Glomerular Ang II content and Ang I/II expression were determined by ELISA and Western blot, respectively. As shown in Figure 4a, the administration of 12(S)-HETE significantly increased glomerular Ang II content compared to the vehicle alone (*p* < 0.01). Similarly, by Western blot, it was revealed that 12(S)-HETE treatment upregulated Ang I/II levels in glomeruli compared to the control (*p* < 0.01; Figure 4b). Next, the effect of 12-LO on Ang II level was detected under diabetic conditions. The content of Ang II (Figure 4c) and Ang I/II (Figure 4d) in glomeruli was higher in type-2 diabetic rats compared to control rats (*p* < 0.01). As expected, CDC treatment significantly decreased Ang II content and Ang I/II expression, compared to those without CDC treatment (*p* < 0.05). These results suggest that Ang II level in glomeruli was activated in type-2 diabetic rats, and this activation was partially attenuated by 12-LO inhibition.

### 2.6. 12(S)-HETE Induced the Expression of AT1R and AT1R-Associated Protein in Rat Mesangial Cells and Glomeruli

**Figure 5:**
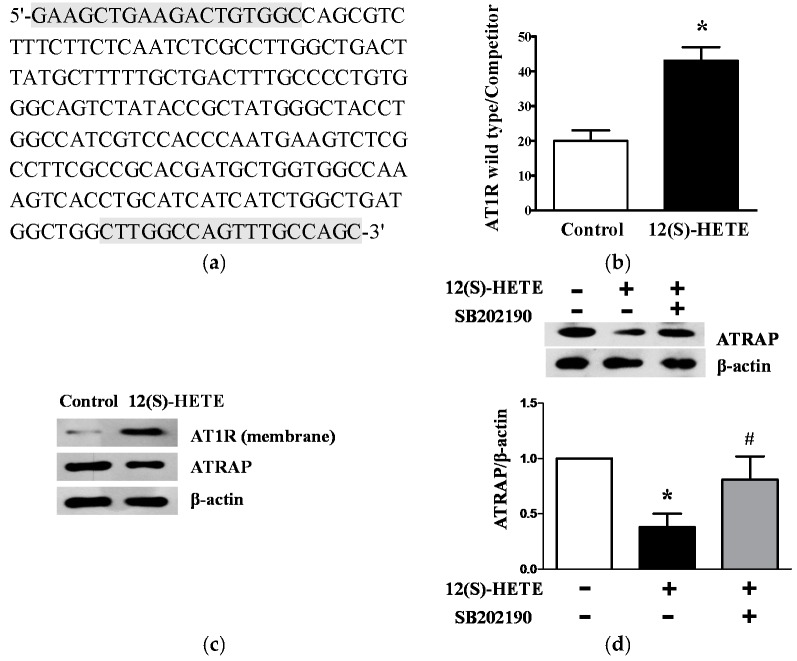
Effect of 12(S)-HETE on AT1R and ATRAP expression in rat glomeruli and MCs. (**a**) The nucleotide sequence of AT1R competitors. The nucleotides used as primers for RT-PCR are indicated by gray boxes; (**b**) Bar graph quantification for glomerular AT1R mRNA expression of control and 12(S)-HETE infusion rats was determined by competitive RT-PCR; *n* = 10 in each group; (**c**) Glomerular membrane AT1R and ATRAP expression of control and 12(S)-HETE infusion rats was determined by Western blot; *n* = 10 in each group; (**d**) Rat MCs were stimulated with 12(S)-HETE and/or SB202190, and ATRAP expression was determined by Western blot. Data are presented as the mean ± SD of three independent experiments; * *p* < 0.01 *vs.* the control; ^#^ *p* < 0.05 *vs.* 12(S)-HETE.

Next, the direct effects of 12(S)-HETE on glomerular AT1R levels were assessed by quantitative competitive transcription-polymerase chain reaction (RT-PCR). An AT1R deletion mutant co-amplified with the endogenous gene was used as the internal control (Figure 5a). As shown in Figure 5b, a significant increase in glomerular AT1R mRNA expression was observed in rats with 12(S)-HETE infusion compared to the control (*p* < 0.01), indicating that 12(S)-HETE could directly increase AT1R expression. Similarly, a significant increase in AT1R protein level was observed in rat glomeruli following 12(S)-HETE infusion compared to the control (Figure 5c). ATRAP functions as a molecular Ang II-induced transduction signal by enhancing AT1R internalization. Interestingly, glomerular ATRAP protein expression was significantly reduced after 12(S)-HETE infusion (Figure 5c).

In order to examine the possible signal pathway of 12(S)-HETE, serum-depleted MCs were treated with 12(S)-HETE (10^−7^ mol/L) with or without p38 mitogen-activated protein kinase (MAPK) inhibitor SB202190 (10^−6^ mol/L). The 12(S)-HETE stimulation alone significantly decreased ATRAP protein expression compared to the control (*p* < 0.01). In the presence of SB202190, 12(S)-HETE-induced ATRAP reduction was clearly attenuated (*p* < 0.05; Figure 5d), indicating that the p38 MAPK pathway was involved in mediating the 12-LO induced decrease in ATRAP.

### 2.7. Effect of 12(S)-HETE and Insulin on AT1R Expression in Rat MCs

**Figure 6:**
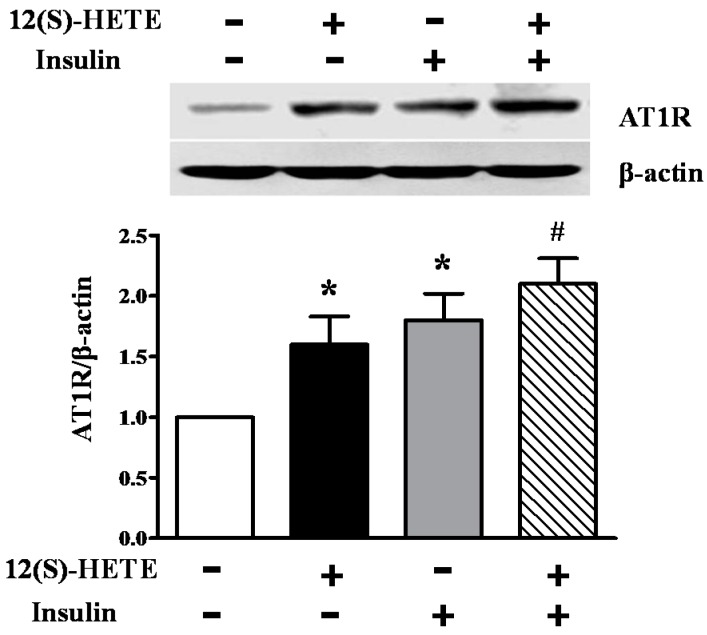
Effect of 12(S)-HETE and insulin on AT1R expression in rat MCs. Cultured rat MCs were stimulated with 12(S)-HETE or insulin for 24 h, and AT1R protein level was determined by Western blot. Data are presented as the mean ± SD of three independent experiments; * *p* < 0.01 *vs.* the control; ^#^ *p* < 0.05 *vs.* 12(S)-HETE or insulin.

In order to investigate the role of 12-LO activation and insulin on AT1R expression, MCs were treated with 12(S)-HETE (10^−7^ mol/L) and/or insulin (10^−6^ mol/L) for 24 h. As shown in Figure 6, although both 12(S)-HETE and insulin significantly increased AT1R protein levels in rat MCs (*p* < 0.01), the combination of 12(S)-HETE and insulin demonstrated a synergetic effect on AT1R upregulation, compared to each sole stimulation alone (*p* < 0.05; Figure 6).

### 2.8. Effect of 12-LO on AT1R and ATRAP Expression in Glomeruli of Diabetic Rats

**Figure 7:**
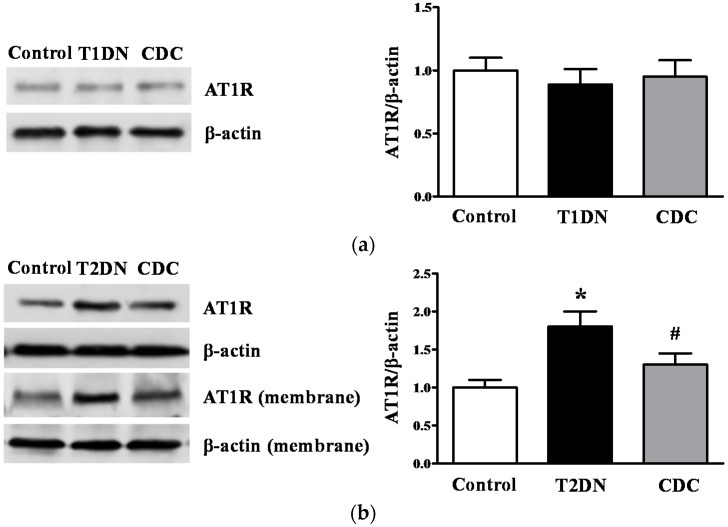

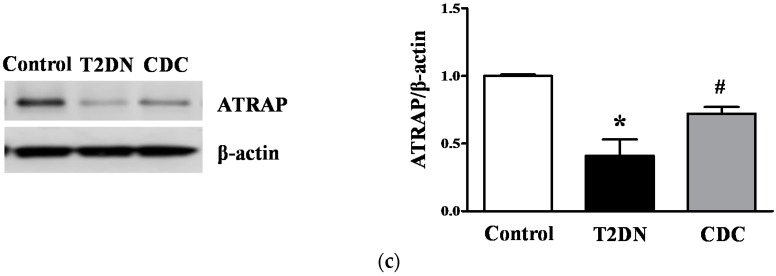
Effect of 12-LO on the AT1R and ATRAP protein expression in glomeruli of type-1 and type-2 diabetic rats. Glomerular AT1R and ATRAP expression was determined by Western blot. (**a**) AT1R protein expression in glomeruli of control, type-1 diabetic rats and CDC-treated rats; *n* = 10 in each group; (**b**) total AT1R and membrane AT1R protein expression in glomeruli of control, type-2 diabetic rats and CDC-treated rats; *n* = 20 in each group; (**c**) ATRAP protein expression in glomeruli of control, type-2 diabetic rats and CDC-treated rats; *n* = 20 in each group. Data are presented as the mean ± SD of three independent experiments; * *p* < 0.01 *vs.* the control; ^#^ *p* < 0.05 *vs.* T2DN.

A slightly decreased AT1R expression was observed in glomeruli in type-1 diabetic rats compared to the control, but this decrease did not reach statistical significance; and 12-LO inhibition did not alter AT1R protein expression (Figure 7a). As shown in Figure 7b, total AT1R protein and membrane AT1R protein expression in glomeruli in type-2 diabetic rats significantly increased compared to the control (*p* < 0.01); and CDC treatment ameliorated AT1R elevation induced by type-2 diabetes (*p* < 0.05). As expected, ATRAP decreased in type-2 diabetic rat glomeruli compared to the control (*p* < 0.01; Figure 7c); but this abnormality was attenuated by CDC treatment (*p* < 0.05, Figure 7c).

## 3. Discussion

In this study, we utilized a nongenetic type-2 diabetic rat model feeding a high fat diet and administrating a low dose of STZ (35 mg/kg), which is characterized by insulin resistance and partial β-cell failure [31]. Besides, we also used type-1 diabetic mice (receiving 50 mg/kg of STZ intraperitoneally on five consecutive days) and rat (65 mg/kg) models by one single injection of high dose STZ, in which a high percentage of endogenous β-cells were destroyed, thus resulting in little endogenous insulin production [30,32]. These models of type-1 and type-2 diabetes are similar to human diabetes and nephropathy, where multiple pathophysiological factors act synergistically to damage kidney [30,31,32].

Studies proved that 12-LO expressions increased significantly in type-1 and type-2 diabetic rat glomeruli compared to control rats [20,23]. It is well known that CDC treatment should mainly only block 12-LO activity and reduce 12(S)-HETE levels. Xu *et al.* demonstrated that relative short-term CDC treatment did not affect glomerular 12-LO expression in type-2 diabetic conditions. Interestingly, glomerular 12-LO increments were significantly attenuated by relative long-term CDC treatment [20]. This is a beneficial effect of persistent CDC treatment. In this study, we observed that 12(S)-HETE levels increased significantly in diabetic glomeruli compared to the control, and these changes were significantly ameliorated by CDC treatment, thus confirming that CDC used in this study is specific and effective.

In the current study, we demonstrated that the 12-LO inhibitor, CDC, can ameliorate the progression of MAU in type-2 diabetic rats, but not in type-1 diabetic rats. Similarly, the deletion of 12-LO exerts no effect on MAU in type-1 diabetic mice. These results are consistent with a previous study, in which 12-LO specific inhibitor, *N*-benzyl-*N*-hydroxy-5-phenyl pentanamide (BHPP), could not inhibit albuminuria in STZ-induced type-1 diabetes mellitus [22], indicating that the inhibition of 12-LO exerts different effects on the progression of MAU in type-1 and type-2 diabetes.

MAU has been recognized as the first sign of nephropathy and a predictor of kidney disease progression in patients with diabetes [33]. Subjects with MAU have lower insulin sensitivity and higher fasting insulin, compared to subjects without MAU; while increased insulin sensitivity is related to decreased MAU prevalence [34]. IR demonstrated a strong and independent relationship with MAU in patients with type-2 diabetes [35]. The mechanism of hyperinsulinemia, which leads to increased MAU levels, could be partly explained by the imbalance between filtered load and the tubular re-absorption of albumin [36]. In this study, we have proven that 12-LO inhibition could ameliorate MAU by influencing IR.

Several studies have suggested that the 12-LO pathway is related to the pathophysiological progression of IR [26,37,38]. In our study, we explored several parameters related to IR, such as fasting insulin level, ISI and AMPK under type-2 diabetic conditions. AMPK activity is involved in insulin-dependent glucose uptake and insulin signaling [39]. The AMPK activation in response to calorie restriction and regular exercise increases skeletal muscle fatty oxidation and insulin sensitivity and decreases metabolic disorders [40]. In the current study, we found that increased fasting insulin levels, decreased insulin sensitivity and reduced p-AMPK levels in type-2 diabetic rats were significantly attenuated by CDC treatment; which further supports the notion that 12-LO inhibition can ameliorate the progression of IR in type-2 diabetic conditions. A previous study revealed that fasting insulin level in WT + STZ mice is lower than 12-LOKO + STZ mice on Days 21 and 28 after STZ injection, and 12-LO elimination did not alter basal or glucose-stimulated islet insulin secretion *in vitro* [32]; suggesting that 12-LO inhibition is resistant to diabetes induction in different ways: 12-LO inhibition exerts a protective effect on pancreatic β-cell damage in type-1 diabetes and plays a pivotal role in the amelioration of IR in type-2 diabetes.

Skeletal muscle is the predominant insulin-responsive peripheral site of glucose metabolism and exerts an important role in maintaining systemic glucose homeostasis [41,42]; therefore, we detected the insulin signaling pathway in skeletal muscle at molecular levels. It is well known that the proximal insulin signaling enzymes IRS-1 and downstream targets Akt play a pivotal role in the insulin signaling pathway. IRS-1 is the key adaptor molecule involved in muscle mediating signal transduction governing the metabolic effect of insulin [43], and Akt mediates skeletal muscle glucose disposal in biological response to insulin [44]. In this study, we found that reduced p-IRS-1 and p-Akt levels in skeletal muscle under the type-2 diabetic condition were evidently ameliorated by CDC treatment, thus demonstrating that 12-LO activation regulates IR through the insulin signaling pathway in the type-2 diabetic model.

It is well known that RAS inhibitors, ACEI or ARB, can increase insulin sensitivity and diminish MAU in type-2 diabetes [11,12,13]. Nowadays, normalizing blood glucose levels and inhibiting Ang II activity with RAS inhibitors, such as ACEI or ARB, remain a standard treatment of DN. The 12-LO pathway is activated in diabetes mellitus with increased 12(S)-HETE. In order to analyze the direct effect of 12(S)-HETE on RAS, we treated rats and rat MCs with 12(S)-HETE and found that this treatment significantly increased Ang II levels and AT1R expression. Notably, CDC treatment significantly reduced Ang II and AT1R expression in glomeruli of type-2 diabetic rats. As expected, in this study, we observed that both the 12-LO lipid product 12(S)-HETE and insulin can increase AT1R expression in cultured rat MCs; and importantly, we also found a significant synergetic effect in combining 12(S)-HETE and insulin on AT1R expression, compared to the effects when observed separately. A previous study revealed that AT1R expression is upregulated by insulin in cultured vascular smooth muscle cells [45], further indicating the involvement of AT1R in IR.

The expressions of AT1R was upregulated in type-2 diabetic glomeruli [20], but was downregulated in type-1 diabetic glomeruli [29]. Consistent with previous studies, we found that AT1R expression increased in type-2 diabetic glomeruli, but this did not change in type-1 diabetic glomeruli. Moreover, CDC treatment only decreased the expression of AT1R and MAU in type-2 diabetic rats, but not in type-1 diabetic rats.

Multiple agonists other than Ang II have been shown to modulate AT1R expression, such as ATRAP, which functions as a negative regulator by enhancing the internalization of AT1R. ATRAP is recognized as an interacting molecule with the AT1R carboxyl-terminal domain, which is involved in the control of AT1R internalization. ATRAP has been reported to suppress Ang II-mediated pathological responses *in vivo* and *in vitro* by promoting the constitutive internalization of AT1R [46]. Previous studies have also suggested a functional interplay between AT1R and 12-LO [10,18,20,21,30]. Therefore, we hypothesized that 12-LO inhibition could lead to the downregulation of AT1R via modulating ATRAP; and thereby ameliorate IR. In this study, we investigated the role of 12(S)-HETE on ATRAP expression, and found that ATRAP obviously decreased in rat glomeruli after 12(S)-HETE infusion, compared to the control. Compared to control rats, ATRAP expression was decreased in type-2 diabetic rats; but this reduction was drastically attenuated by 12-LO inhibition. In order to explore the underlying mechanism, we also treated rat MCs with 12(S)-HETE and p38 MAPK inhibitor, SB202190. Fascinatingly, the ATRAP reduction induced by 12(S)-HETE was clearly attenuated in the presence of SB202190, suggesting that the p38 MAPK pathway was involved in the 12-LO-ATRAP interaction. Considering that ATRAP is an endogenous inhibitor of AT1R signaling, we concluded that AT1R could be regulated by 12-LO-ATRAP interaction via the p38 MAPK pathway.

In summary, AT1R upregulation in type-2 diabetic glomeruli is probably associated with IR progression. Attenuating IR by inhibiting 12-LO activation to downregulate glomerular AT1R levels prevented the development of MAU in type-2 diabetes. However, further studies are required to comprehensively evaluate the underlying mechanisms.

## 4. Materials and Methods

### 4.1. Materials

The 12-LO inhibitor, CDC and 12(S)-HETE were obtained from Biomol (Plymouth Meeting, PA, USA); p21, p27, AT1R, AMPK, p-AMPK, Ang I/II and ATRAP antibodies were obtained from Santa Cruz Biotechnology (Santa Cruz, CA, USA); IRS-1, p-IRS-1 (Tyr612), Akt and p-Akt (Tyr308) antibodies and horseradish peroxidase-conjugated secondary antibodies were obtained from Cell Signaling (Beverly, MA, USA); β-actin antibody, STZ and insulin were obtained from Sigma (St. Louis, MO, USA); the plasma membrane protein extraction kit and SuperSignal chemiluminescent reagents were obtained from Pierce (Rockford, IL, USA); RPMI 1640 medium and fetal bovine serum (FBS) were obtained from Gibco BRL (Grand Island, NY, USA); SB202190 was obtained from Calbiochem (La Jolla, CA, USA); RNA STAT-60 reagent was obtained from Tel-Test (Friendswood, TX, USA); the RT-PCR kit was obtained from Ambion Inc. (Austin, TX, USA); the Urinary Albumin ELISA Kit was obtained from Exocell Inc. (Philadelphia, PA, USA); the Ang II ELISA kit and the 12(S)-HETE ELISA kit were obtained from Assay Designs (Ann Arbor, MI, USA); the Insulin RIA Kit was obtained from Linco Research Inc. (St. Charles, MO, USA); the osmotic mini-pump (Alzet Model 1002) was obtained from Durect Corp. (Cupertino, CA, USA).

### 4.2. Cell Cultures

Primary MCs from Sprague-Dawley rats were isolated, as previously described [23], and were cultured in RPMI 1640 supplemented with 10% FBS at 37 °C in 5% CO_2_. Cells between five and eight passages were used in the current study. For Western blot, MCs were plated in 100-mm dishes and cultured in RPMI medium containing normal glucose (5.6 mM, NG) with 20% FBS. Cells were allowed to grow for five days to confluence, followed by serum starvation with the NG medium containing 0.2% bovine serum albumin for 48 h. Thereafter, the medium was changed to NG, NG + insulin (10^−6^ mol/L), NG + 12(S)-HETE (10^−7^ mol/L) or NG + insulin (10^−6^ mol/L) + 12(S)-HETE (10^−7^ mol/L). MCs were harvested 24 h later. In another experiment, serum-depleted MCs were pretreated with the p38 MAPK inhibitor, SB202190 (10^−6^ mol/L), for 30 min, and stimulated with 12(S)-HETE (10^−7^ mol/L) for 24 h. The concentrations of insulin, 12(S)-HETE and SB202190 used in this study were determined based on preliminary experiments.

### 4.3. 12(S)-HETE Infusion by the Osmotic Mini-Pump

Twenty male Sprague-Dawley rats weighing 180–200 g were used in this study. The rats were randomly assigned to receive vehicle (normal saline or ethanolamine; *n* = 10) or 12(S)-HETE (infusion rate, 1 mg/kg/day; *n* = 10) by osmotic mini-pumps. The dose of 12(S)-HETE was based on our previous study [21]. The mini-pumps infused 12(S)-HETE and ethanolamine (control) continuously for seven days. Systolic blood pressure was measured by tail-cuff plethysmography, and blood glucose was measured by glucometer daily. Then, rats were sacrificed, and glomeruli were isolated and stored at −70 °C for further study.

### 4.4. Induction of Type-1 and Type-2 Diabetes

All animal studies were conducted under a protocol (project identification code: 2014-283, date: 06 January 2014) approved by the committee for the care and use of laboratory animals of the First Hospital of Jilin University (Changchun, China).

Thirty two male Sprague-Dawley rats, weighing 200–220 g, were used for the type-1 diabetes model. Ten rats were injected with diluents (control, *n* = 10), while 22 rats were intraperitoneally administered with 65 mg/kg of STZ. Fasting blood glucose levels were measured three days after STZ injection. Fasting glucose >16.7 mmol/L was determined as diabetes. Then, diabetic rats were randomly assigned into two groups. One group was left untreated (T1DN, *n* = 10), and the other 10 diabetic rats were treated with CDC (8 mg/kg) for six weeks (CDC, *n* = 10). Male C57BL/6 mice and knockout (LOKO) mice (Jackson Laboratories, Bar Harbor, ME, USA) weighing 22–25 g were used to establish the type-1 diabetes model. Ten of each kind of mice were intraperitoneally administered with a total of 250 mg/kg of STZ (50 mg/kg of STZ intraperitoneally on 5 consecutive days, *n* = 20). Mice were injected with diluents served as the control (*n* = 20). Fasting blood glucose levels were measured three days after STZ injection to confirm the development of diabetes. Fasting glucose >16.7 mmol/L was determined as diabetes. Wild-type and 12-LOKO C57BL/6 mice were divided into four groups: wild-type control (WT, *n* = 9), 12-LOKO (LOKO, *n* = 9), STZ-induced wild type-1 diabetes (WT + STZ, *n* = 9) and STZ-induced 12-LOKO type-1 diabetes (LOKO + STZ, *n* = 9).

Sixty four male Sprague-Dawley rats, weighing 200–220 g, were used for the type-2 diabetes model. Rats were randomly assigned to either regular rat chow (*n* = 20) or a 60% high fat diet (Research Diets, D12492, New Brunswick, NJ, USA; *n* = 44). After six weeks on their respective diets, rats fed with the high fat diet intraperitoneally received low-dose STZ (35 mg/kg, *n* = 44); rats fed with regular chow (control, *n* = 20) were intraperitoneally injected with citrate buffer alone. Fasting blood glucose levels were measured three days after STZ injection to confirm the development of diabetes, and fasting blood glucose >16.7 mmol/L was defined as the threshold for diabetes. Rats fed with high fat diet were divided into a low-dose STZ treatment group (T2DN, *n* = 20) and a low-dose STZ + CDC treatment group (8 mg/kg; CDC, *n* = 20). The vehicle (sesame oil) or CDC was subcutaneously injected in the hind leg three times weekly on alternate days after the induction of diabetes. The CDC dose was based on the previous study [20].

Animals were sacrificed after six weeks from diabetes onset. Kidney cortical tissues and skeletal muscles from the legs were stored at −70 °C. Additional kidney cortical tissues were fixed in 10% formalin for histologic evaluation. Twenty four-hour urinary albumin excretion was determined by ELISA, and fasting blood glucose was measured by a glucometer. Fasting insulin level and ISI were examined as key parameters of IR. Fasting insulin level was measured by a specific Insulin RIA Kit. ISI was chosen to predict changes in insulin sensitivity: ISI = 1/(fasting insulin × fasting glucose) [47]. The distribution of ISI is skewed; thus, the natural logarithm form of ISI was used for statistical analysis.

### 4.5. Cell Membrane and Glomeruli Isolation

The plasma membrane from glomeruli was specifically extracted from tissues using a Plasma Membrane Extraction Kit (Pierce, Rockford, IL, USA), according to the manufacturer’s protocol. Glomeruli were isolated by sieving, based on a previous study [48]. The purity of the glomerular preparation was >98%, as determined by light microscopy.

### 4.6. Measurement of 12(S)-HETE and Ang II

Glomeruli were lysed in sodium dodecyl sulfate (SDS) sample buffer. Lysates were centrifuged at 12,000 rpm for 15 min at 4 °C, and the supernatant was stored at −70 °C. Protein content was measured with the modified Lowry method. 12(S)-HETE and Ang II levels were quantified by ELISA using a commercial kit according to the manufacturer’s instructions.

### 4.7. Competitive RT-PCR

Total RNA from glomeruli was extracted using RNA STAT-60 reagent, according to the manufacturer’s instructions; and cDNA was synthesized with 1 μg of RNA using MuLV RT and random hexamers. Sense (5′-GAAGCTGAAGACTGTGGC-3′) and antisense (5′-GCTGGCAAACTGGCCAAG-3′) primers were used to amplify AT1R (317 bp). The AT1R competitor cDNA (212 bp) was used as the internal standard. The AT1R competitor cDNA (a portion of the cDNA was deleted) was designed with the same sequence as the target cDNA to enable efficient priming. For AT1R mRNA expression levels, competitive RT-PCR was used, as previously described [20]. PCR products were separated on agarose gels and stained with ethidium bromide. RNA quantity was expressed relative to the competitor fragments.

### 4.8. Western Blot Analysis

Rat MCs, glomeruli and skeletal muscles were lysed in SDS sample buffer. The lysate was centrifuged at 12,000 rpm for 10 min at 4 °C, and the supernatant was stored at −70 °C. Fifty micrograms of the protein of each sample were loaded on an 8%–12% SDS-PAGE, transferred to a membrane and immunoblotted with antibodies to AT1R (1:1000), p-AMPK (1:1000), AMPK (1:1000), IRS-1 (1:1000), p-IRS-1 (1:1000), Akt (1:1000), p-Akt (1:1000), p21 (1:1000), p27 (1:1000), Ang I/II (1:1000), ATRAP (1:1000) and β-actin (1:3000). The blots were scanned using a densitometer, and bands were quantitated with Quantitation One software (Bio-Rad Laboratories, Hercules, CA, USA).

### 4.9. Histological Evaluation

In order to perform PAS staining, renal cortical slices were fixed in 10% neutral buffered formalin prior to paraffin embedding, processed in the standard manner, and 5 μm sections were utilized. Slides were deparaffinized, hydrated and oxidized with periodic acid for 12 min. After washing, the slides were stained with Schiff dye for 20 min and counterstained with hematoxylin for one minute. Glomeruli were examined via light microscopy at 400× magnification. Glomerular volume (*V*_G_) was calculated using a computer-assisted color image analyzer Image-Pro (Version 2.0; Media Cybernetics, Silver Spring, MD, USA) according to the method of Weibel [49]. *V*_G_ = β/k × (area)^3/2^, where β = 1.38 (the shape coefficient for the spheres) and k = 1.1 (the size distribution coefficient).

### 4.10. Statistical Analysis

Statistical analysis was performed with PRISM software (Graph Pad, San Diego, CA, USA). Paired Student’s *t*-tests were used to compare two groups or ANOVA with Dunnett’s *post*-test for multiple groups. All data are presented as the mean ± SD. A *p*-value of <0.05 was considered to indicate statistically-significant differences.

## Abbreviations

12-LO	12-lipoxygenase
DN	Diabetic nephropathy
T2DN	Type-2 diabetic nephropathy
Ang II	Angiotensin II
AT1R	Angiotensin II type 1 receptor
MAU	Microalbuminuria
ATRAP	Angiotensin II type 1 receptor-associated protein
MAPK	Mitogen-activated protein kinase
IR	Insulin resistance
RAS	Renin-angiotensin system
ACEI	Angiotensin-converting enzyme inhibitor
ARB	Angiotensin II type 1 receptor blocker
12(S)-HETE	12(S)-hydroxyeicosatetraenoic acid
CDC	Cinnamyl-3,4-dihydroxy-α-cynanocinnamate
AMPK	AMP-activated protein kinase
IRS	Insulin receptor substrate
ELISA	Enzyme-linked immunosorbent assay
RIA	Insulin radioimmunoassay
MCs	Mesangial cells
STZ	Streptozotocin
FBS	Fetal bovine serum
RT-PCR	Reverse transcription–polymerase chain reaction
ISI	Insulin sensitivity index
PAS	Periodic acid-Schiff
BHPP	*N*-benzyl-*N*-hydroxy-5-phenylpentanamide
